# The integrated pathway of TGFβ/Snail with TNFα/NFκB may facilitate the tumor-stroma interaction in the EMT process and colorectal cancer prognosis

**DOI:** 10.1038/s41598-017-05280-6

**Published:** 2017-07-07

**Authors:** Hui Li, Anjing Zhong, Si Li, Xianwen Meng, Xue Wang, Fangying Xu, Maode Lai

**Affiliations:** 1 Department of Pathology, School of Medicine, Zhejiang University, Hangzhou, 310058 China; 2 Key Laboratory of Disease Proteomics of Zhejiang Province, Hangzhou, 310058 China; 3 Department of Bioinformatics, State Key Laboratory of Plant Physiology and Biochemistry, College of Life Sciences, Zhejiang University, Hangzhou, 310058 China; 4 China Pharmaceutical University, Nanjing, 320100 China

## Abstract

Substantial evidence has shown that epithelial-mesenchymal transition (EMT) plays critical roles in colorectal cancer (CRC) development and prognosis. To uncover the pivotal regulators that function in the cooperative interactions between cancer cells and their microenvironment and consequently affect the EMT process, we carried out a systematic analysis and evaluated prognosis in CRC specimens. Tumor buds and their surrounding stroma were captured using laser microdissection. We used gene expression profiling, bioinformatics analysis and regulatory network construction for molecular selection. The clinical significance of potential biomarkers was investigated. We identified potential EMT biomarkers, including *BGN, MMP1*, *LGALS1, SERPINB5*, and *TM4SF4*, all of which participated in the integrated pathway of TGFβ/Snail with TNFα/NFκB. We also found that *BGN*, *MMP1*, *LGALS1*, *SERPINB5* and *TM4SF4* were related to CRC patient prognosis. Patients with higher expression of these individual potential biomarkers had poorer prognosis. Among the identified biomarkers, BGN and TM4SF4 are reported, for the first time, to probably be involved in the EMT process and to predict CRC prognosis. Our results strongly suggest that the integrated pathway of TGFβ/Snail with TNFα/NFκB may be the principal axis that links cancer cells to their microenvironment during the EMT process and results in poor prognosis in CRC patients.

## Introduction

In the EMT process, cells lose their epithelial characteristics such as cell polarity and gradually obtain mesenchymal characteristics; this process is accompanied by the down-regulation of the epithelial biomarker E-cadherin (CDH1)^1^. Tumor cells undergoing EMT become mobile and invasive, thus allowing for their dissemination. Substantial evidence has revealed that cancer cells and the tumor microenvironment cooperatively regulate the EMT program and cancer metastasis^2–5^. In addition, cancer cells and their microenvironment both can influence patient prognosis or by means of cooperation^6–8^. Previous studies have uncovered several EMT biomarkers including growth factors, such as TGFβ, Wnt, Hedge hog, Notch, RhoA, Ras, EGF and HGF; cytokines, such as TNFα, HIF1α, NF-κB and Akt; transcriptional factors, such as Snail, Twist, Zeb1, Slug and Stat3; and other markers, such as MMPs, Goosecoid, BMPs, PI3K and Met^9–11^. Although many EMT biomarkers have been identified, the mechanism of the cooperative regulation between cancer cells and their microenvironment and the effect on the EMT process remains unclear. There is substantial work to be done to uncover these mechanisms and their effects on the behavior of cancer, and to discover additional therapeutic targets.

Tumor buds, defined as a single cancer cell or small clusters of up to five cells at the invasive front in several types of cancers, including colon, breast and pancreatic cancer, have long attracted attention as a prognostic marker for cancer^12–14^. Tumor buds are non-proliferating, non-apoptotic, and highly aggressive subpopulations of cancer cells with migratory and invasive characteristics. Tumor budding is thought to result from the EMT program even though it is not equivalent to EMT^15^. Studies have also verified that tumor budding formation shares common signaling pathways with the EMT process, such as Wnt and TGFβ signaling pathways^16^. At the molecular level, the epithelial biomarker CDH1 is downregulated, and the mesenchymal biomarkers N-cadherin (CDH2) and fibronectin (FN1) are upregulated in tumor buds, thus suggesting that tumor buds have mesenchymal characteristics. Therefore, tumor budding is an ideal model to study EMT and uncover EMT biomarkers. Because the critical regulatory signaling pathway responsible for the effects of the interactions between cancer cells and their microenvironment on EMT and CRC prognosis remains unknown, tumor buds and the surrounding stroma may be used to reveal this key pathway.

## Results

### Preliminary screening of differentially expressed genes of tumor budding cells and surrounding stromal cells *via* expression profile microarrays

**Figure 1:**
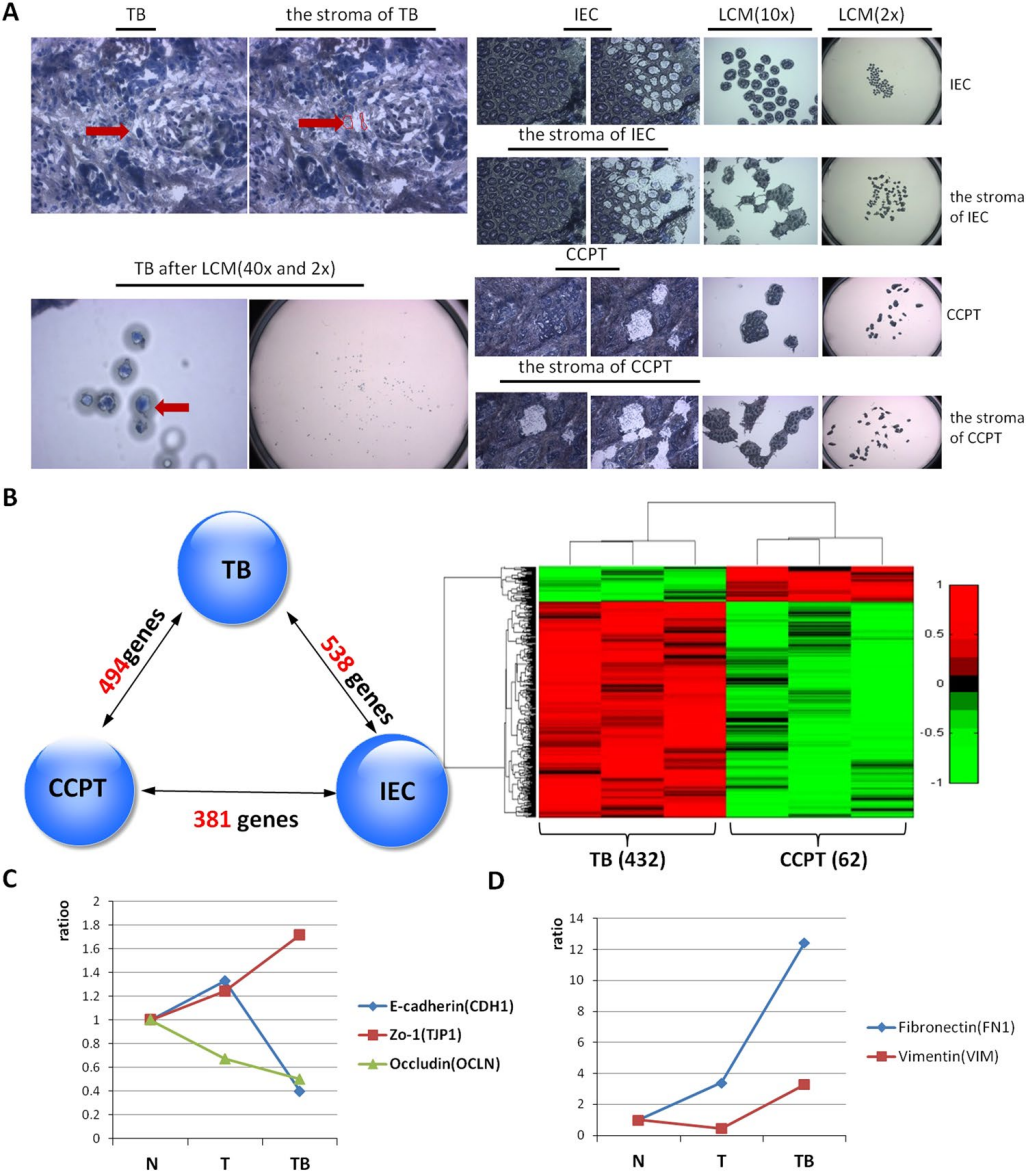
Single-cell capture and gene selection in tumor buds. (**A**) Capture of the tumor buddings (TB) and the surrounding stromal components of tumor buddings (the stroma of TB) with laser capture microdissection, with IEC, CCPT and their stroma as controls. (**B**) Four hundred and ninety-four genes including 432 upregulated genes and 62 downregulated genes that were differentially expressed in the tumor bud samples compared with CCPT, were selected by bioinformatics and statistical analyses (*P* < 0.05; FDR = 0.049773; fold change > 2 & < 0.5). (**C**,**D**) The expression levels of classical epithelial and mesenchymal biomarkers in IEC (N), CCPT (T) and tumor buds (TB). The epithelial markers CDH1 and OCLN were downregulated, and the mesenchymal markers FN1 and VIM were upregulated in the tumor bud samples.

**Figure 2:**
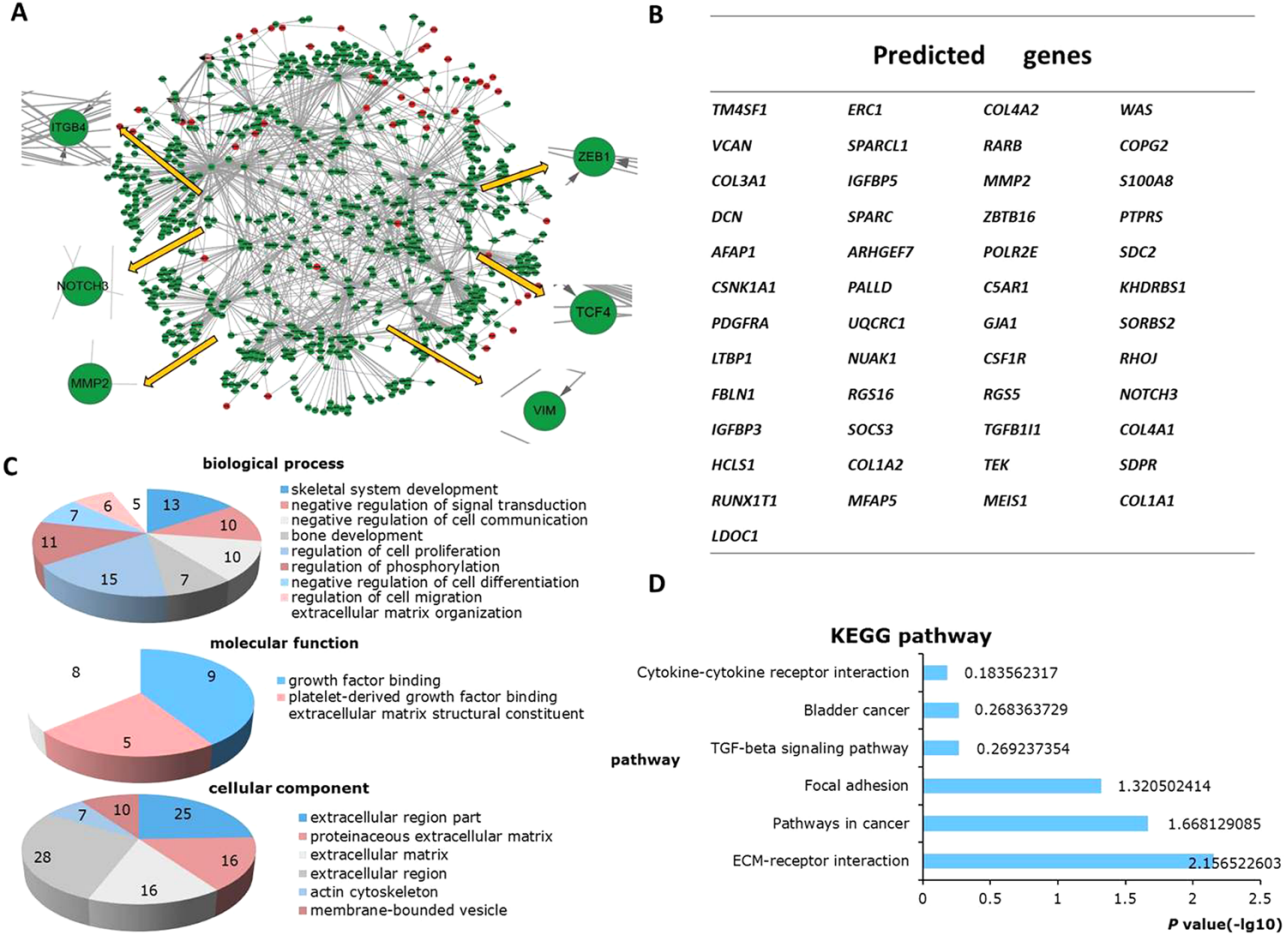
Data mining and functional analysis of differentially expressed genes in tumor bud. (**A**) Potential EMT biomarkers (red spots) selected from tumor the bud samples mapped on a classical EMT-regulator network (green spots) constructed with CFinder (the classical EMT regulators were from an online database)^43^. TCF4, VIM, ZEB1, ITGB3, NOTCH3, and MMP2, are the classical EMT biomarkers that were used to show the reliability of our data mining from the online database. (**B**) Forty-nine genes that functionally interacted with the classical EMT regulators, on the basis of the PPI network are shown in the three-line table. (**C**,**D**) GO and KEGG pathway analyses were used to identify the biological processes, molecular functions, cellular components and signaling pathways of the 49 genes (*P* < 0.05).

We chose three cases for our experiments among fifty CRC cases, on the basis of pathological observation. The selection was based on whether the amount of tumor budding at the invasive front consisted of more than 10 tumor budding cells, as viewed under a 10x microscopic field of the tissue slice. We precisely captured single or clusters of tumor buddings and the microenvironment of tumor budding (TBM) at the invasive front of CRC by using laser capture microdissection. We captured 500 tumor buds and the surrounding stromal components for each case, and RNA was carefully isolated from the samples. After amplification, the cDNA of tumor buds and the surrounding stroma was analyzed with Affymetrix U133 plus 2.0 microarrays. Intestinal epithelial cells (IECs) and cancer cells in the center of the primary tumor (CCPT) and their surrounding stromal components were captured as controls (Fig. 1A). Among forty thousand target genes, 494 differentially expressed genes that passed the false discovery rate (FDR) were selected in the tumor bud cells, including 432 upregulated genes and 62 downregulated genes, compared with CCPT (*P* < 0.05, FDR = 0.049773, fold change > 2 & < 0.5; Fig. 1B). Classical epithelial markers, such as CDH1 and occludin (OCLN), were downregulated in the tumor bud samples compared to IEC and CCPT. In contrast, zo-1 (TJP1) was overexpressed in the tumor bud samples. It is possible that zo-1 is secreted by different types of cells, including cancer cells and fibroblasts, and the activation of Snail in the EMT process may upregulate the expression level of one of the zo-1 isoforms^17^. Mesenchymal markers, including FN1 and vimentin (VIM), were overexpressed in tumor buds compared with IEC and CCPT, thus suggesting an EMT phenotype that was characteristic of the tumor bud cells (Fig. 1C,D). Using principal component analysis, we found that tumor bud cells were situated between the epithelial and mesenchymal cells, and were close to their surrounding stroma (see Supplementary Fig. S1). Then, we constructed an EMT regulatory network of verified EMT biomarkers by using online databases from NCBI (Fig. 2A). We calculated the Pearson correlation coefficients between 494 alternative genes and the verified EMT biomarkers in the regulatory network and mapped the 494 genes in the ready-built EMT regulatory network based on protein-protein interactions (PPI) (Fig. 2A). Forty-nine genes, that had protein level interactions with the verified EMT biomarkers were identified and recognized as potential EMT biomarkers (Fig. 2B). Gene ontology (GO) and KEGG pathway analysis revealed that the 49 genes predominantly participated in the development of the skeletal system, the negative regulation of cell differentiation, and the regulation of cell migration, and the genes were separately involved in ECM-receptor interactions, cancer and TGFβ signaling pathways (Fig. 2C,D).

**Figure 3:**
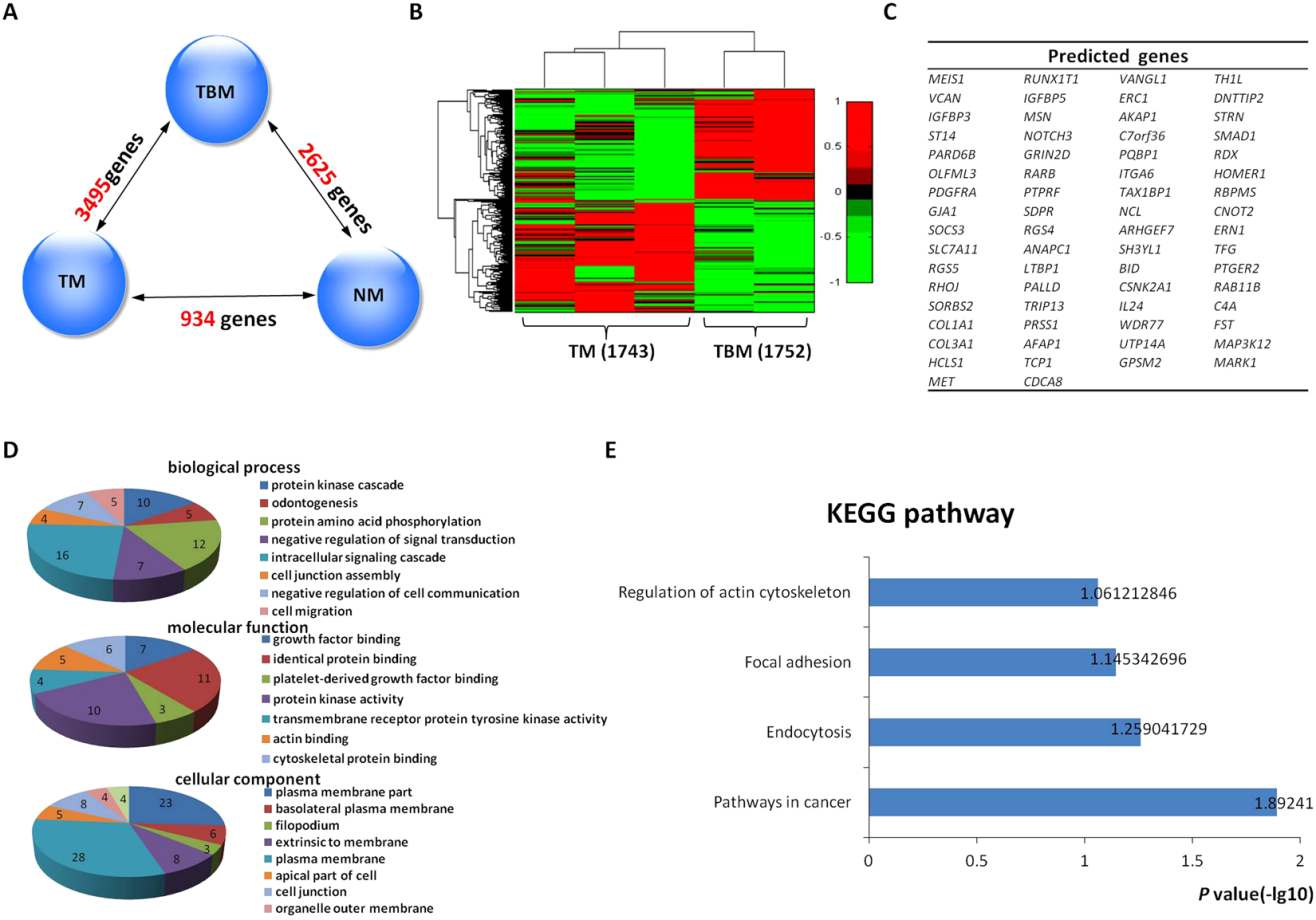
Selection of differentially expressed genes in the stroma cells of the tumor buds and their functional analysis. (**A**,**B**) Differentially expressed genes in each pair of the stromal components of IEC (NM), CCPT (TM) and tumor buds (TBM). The 3,495 differentially expressed genes were selected from TBM, including 1,752 upregulated genes and 1,743 downregulated genes, and are shown in the clustering analysis based on gene-expression levels (fold change > 2 & < 0.5).(**C**,**D** and **E**) Sixty-six genes were revealed through our methods. GO and KEGG pathway analyses were used to identify the biological processes, molecular functions, cellular components and signaling pathways of 66 genes, executed by DAVID 6.7 (*P* < 0.05).

Statistical analysis showed that 3,495 genes were differentially expressed in the microenvironment of tumor buds (TBM) compared with the surrounding stroma of CCPT (TM), including 1,752 upregulated genes and 1,743 downregulated genes (Fig. 3A,B). We further analyzed the data for the stroma of tumor buds by using the same analytic methods used for the tumor budding data. We calculated the correlation coefficients between the 3,495 alternative genes and the verified EMT biomarkers in the regulatory network and mapped these genes in the ready-built EMT regulatory network based on PPI. Sixty-six genes were found to have interactions with classical EMT biomarkers at the protein level, thus suggesting that these genes might directly or indirectly be involved in the EMT program (Fig. 3C). GO analysis showed that 66 genes had biological functions associated with intracellular signal cascades and cell migration and cell junction assembly, and they participated in cell signaling transduction of cancer, endocytosis, focal adhesion and the regulation of actin cytoskeleton, thereby suggesting that these 66 genes might affect EMT development (Fig. 3D,E).

**Figure 4:**
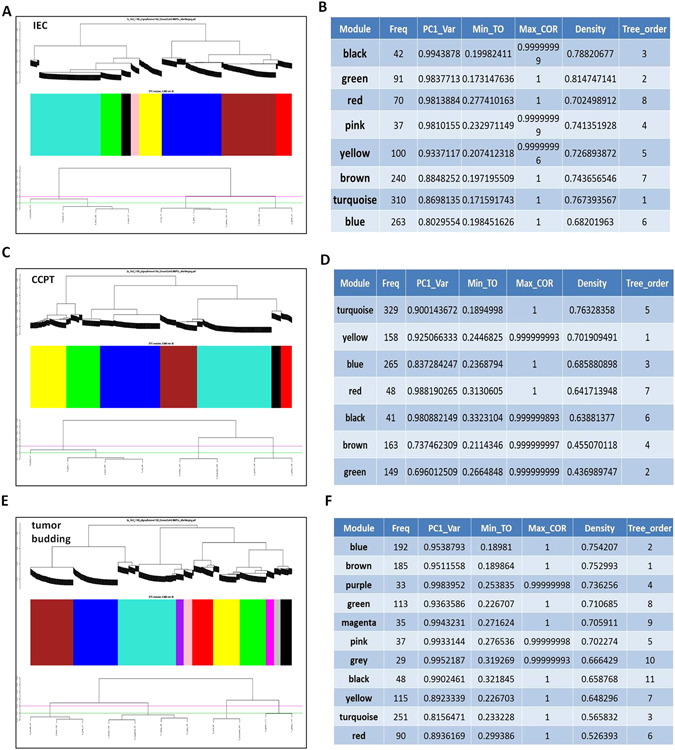
Gene module construction of IEC, CCPT, and tumor buds. (**A**,**C** and **E**) The gene module construction was based on topological overlap. The gene modules are shown in different colors. Each color stands for a gene module in which the genes in the model had the same expression trend and thus might perform the same function at the protein level. (**B**,**D** and **F**) The biological characteristics of the gene module: Freq represents the number of genes enriched in the gene module; PC1_Var represents the variation range of gene module. The larger the variation value, the greater the specificity of the gene module; Density represents the stability of the gene module. The greater the density value, the more conservative of the gene module; Min_To indicates the minimum topological overlap value between genes in the module, which means the minimum correlation coefficient among genes enriched in the gene module, and Max_COR indicates the maximum topological overlap value between genes in the module. The larger the topological overlap value, the greater the correlation among genes in the module. Tree_order represents the ID of the gene module.

Cancer development is a multimodal and multistep progress, and each stage of cancer development has a unique regulated gene set. We constructed gene modules of IEC, CCPT, and tumor buds through weighted gene co-expression network analysis (WGCNA). We calculated the Pearson correlation coefficient across all the differentially expressed genes in each group and derived the topological overlap, which indicated the degree of connection among the genes. We classified the genes that shared the same expression trend on the basis of the topological overlap by average linkage hierarchical clustering. Finally, we obtained the gene modules of the three groups of CRC cases, and the category of gene modules was illustrated with the evolutionary tree (Fig. 4A,C and E). Gene module construction was on the basis of the topological overlap. Gene modules are shown in different colors. Each color stands for a gene module in which the genes in the model have the same expression trend and may perform the same function on protein level. Among these genes, some of them have no strong connections with others are shown in grey. We obtained eight gene modules of IEC, 7 gene modules of CCPT, and 11 gene modules of tumor buds through clustering analysis (Fig. 4A, C and E). The characteristics and parameter values of the gene modules are shown in Fig. 4B, D and F. According to the parameter values of PC1_Var and Density of the gene modules, which the maximum value of PC1_Var and Density stood for the most specific and conservative of gene module, we concluded that the most specific gene module of IEC was shown in black (The value is 0.9943878) and contains 42 genes and the most conservative module was shown in green on the evolutionary tree (The value is 0.814747141, Fig. 4A,B). The most specific gene module of CCPT, shown in red (The value is 0.9943878), contains 48 genes, and the extremely conservative module of CCPT is shown in turquoise (The value is 0.76328358, Fig. 4C,D). The most specific gene module of the tumor buds is shown in purple (The value is 0.9983952), and 33 genes are present in this module. The gene module shown in blue was most conserved on the evolutionary tree (The value is 0.754207, Fig. 4E,F).

We considered the most specific and conservative gene modules of the tumor buds and compared them with those of CCPT and IEC that were likely to be involved in the formation of tumor buds and perhaps even in the EMT process. We executed cross-over compared between gene modules of tumor buds, IEC and CCPT. We rejected gene modules of tumor buds that had the same gene sets with gene modules of IEC and CCPT, and picked out five gene modules that had no overlap of gene sets with the modules of IEC and CCPT. Then we recommended gene sets on the principle of PC1 > 0.90 and density > 0.70, and we obtained five gene sets. The five gene sets of tumor buds were likely to be involved with the core process of tumor budding (see Supplementary Table S1). It implies that the selection of these five gene sets can drive future mechanistic studies in pre-clinical cancer models to verify more EMT biomarkers and CRC prognostic factors. We performed a GO analysis and KEGG pathway analysis on these five gene sets. The results showed that the differentially expressed genes in the five gene sets primarily participate in the biological processes involved in the regulation of locomotion, cell adhesion, biological adhesion, regulation of cell migration, cell motion, cell migration, and response to wounding (see Supplementary Table S2); these genes were also enriched in cancer-related pathways, such as focal adhesions, pathways in cancer, extracellular matrix (ECM)-receptor interactions, the TGFβ signaling pathway and the regulation of the actin cytoskeleton (see Supplementary Table S3). The enrichment analysis clearly showed that the biological activities of these five gene sets were related to the EMT process, thus providing insight into identifying additional EMT biomarkers for future research.

### The preliminary identification of differentially expressed genes

**Figure 5:**
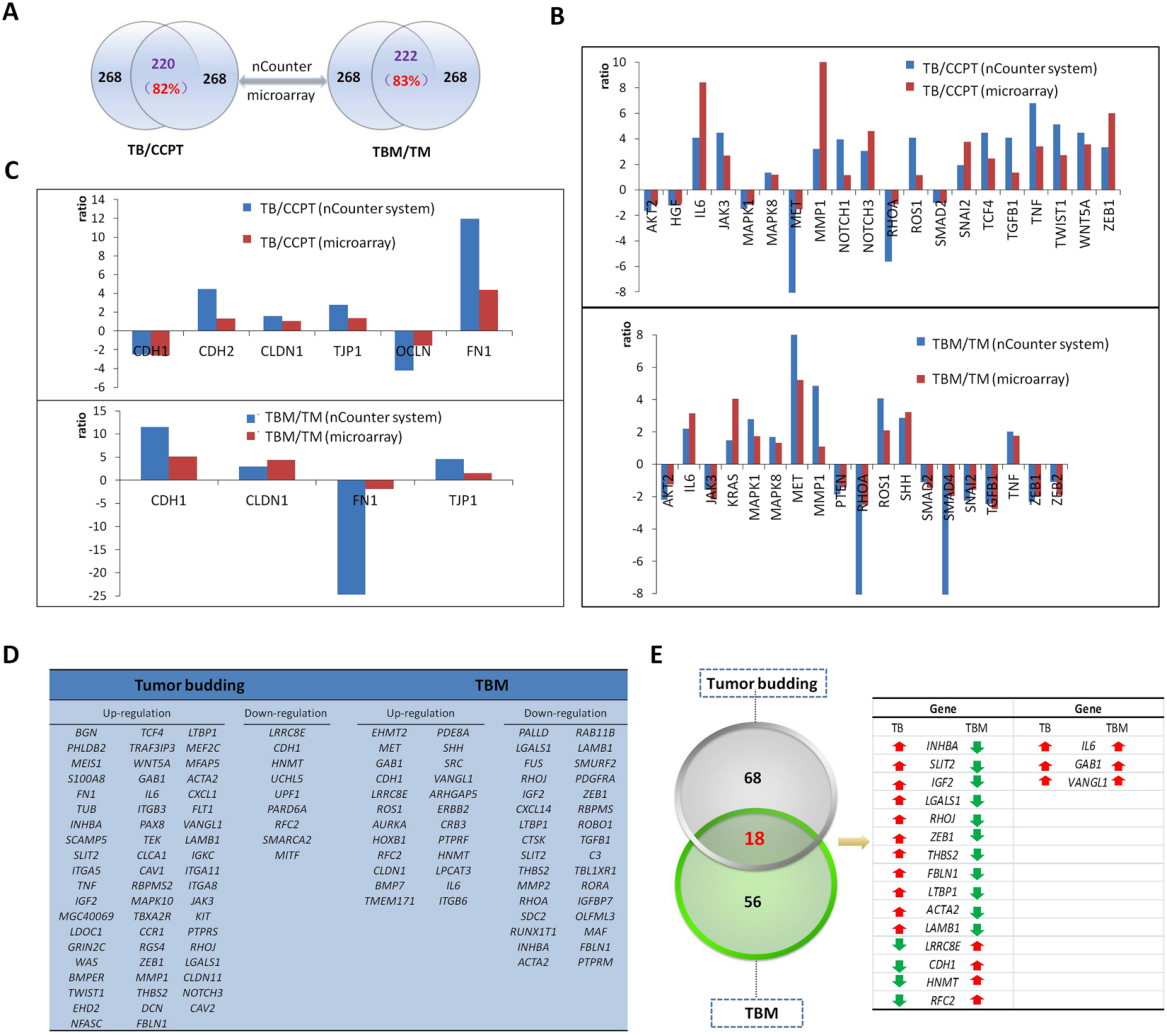
The primary identification of differentially expressed genes with the nCounter system. (**A**) The primary identification results of the 268 candidate genes with the nCounter system. In tumor buds, compared with CCPT, 220 differentially expressed genes had the same expression trend as in the microarray, accounting for 82% of the 268 candidate genes. Additionally, there were 222 genes in TBM that shared the same expression trend as TM, accounting for nearly 83% of the candidate genes. (**B**) The identification of classical EMT regulators in the tumor bud cells and their surrounding stromal cells. (**C**) The identification of classical epithelial and mesenchymal regulators. (**D**) In total, 106 genes, including 59 upregulated and 9 downregulated genes in the tumor buds and 24 upregulated and 32 downregulated genes in TBM, are shown (ratio > 2 & < −2). (**E**) Eighteen of the 106 genes were differentially expressed in both the tumor bud samples and their surrounding mesenchymal cells.

We carefully chose 268 genes, including 202 differentially expressed genes from tumor buds and their surrounding stromal components, according to results from the bioinformatics analyses described above (clustering, GO, KEGG pathway, EMT-regulatory network and gene module analysis). As a control, we collected 66 classical EMT biomarkers for preliminary identification. We precisely captured single or clusters of tumor bud cells and the surrounding stromal component at the invasive front of 4 cases of CRC specimens and detected the expression of the 268 genes in these samples with the nCounter system (see Supplementary Table S4). We normalized the expression levels of candidate genes on the basis of the fluorescence signals of the housekeeping genes *GAPDH* and *ABCF1*. We excluded one case of poor quality and used the 3 other cases for further analysis. We performed *t*-test and ratio calculations and concluded that 220 out of 268 genes (82%) shared the same expression trend in tumor buds, and 222 out of 268 genes (83%) shared the same expression trend in the stromal cells of the tumor buds, as compared with the genes detected by the microarray. The analysis with the nCounter system was in good agreement with the results of the microarray (Fig. 5A). We detected 66 verified EMT biomarkers in the CRC specimens, thus further confirming the accuracy of our approach (Fig. 5B). We then screened out 106 genes from the 268 genes (ratio > 2 & < −2; Fig. 5D). This screening showed that 18 genes were differentially expressed both in the tumor buds and in the microenvironment (Fig. 5E). Genes such as *IL6, GAB1*, and *VANGL1*, were upregulated both in the tumor buds and in the microenvironment. Genes such as *INHBA, IGF2, LGALS1, RHOJ*, and *THBS2* were upregulated in the tumor buds but downregulated in the microenvironment. Other genes such as *LRRC8E, HNMT*, and *RFC2* were downregulated in the tumor buds but upregulated in the microenvironment (Fig. 5E).

### The interactions of tumor buds and the microenvironment in the EMT process

**Figure 6:**
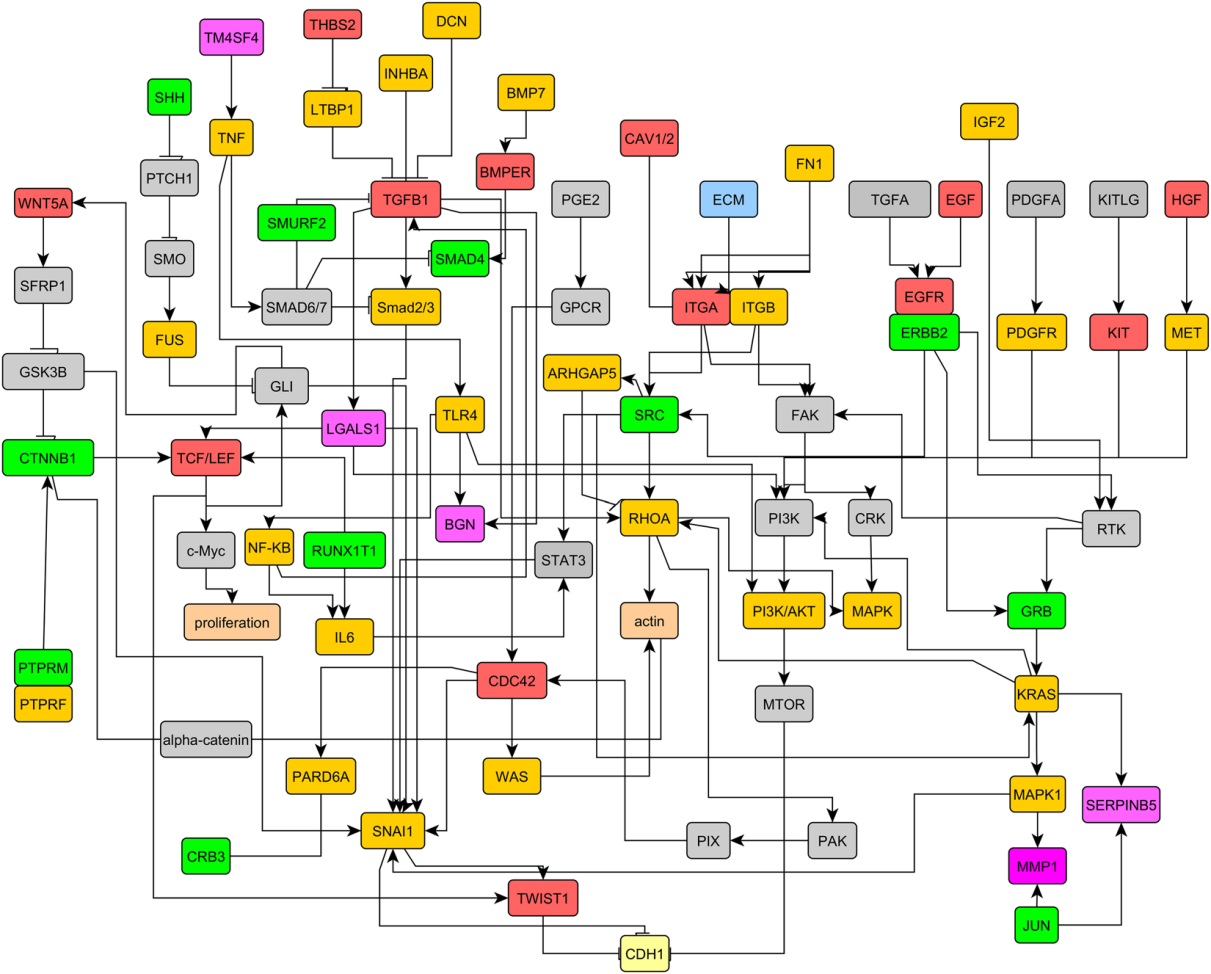
The interaction network of cancer cells and their microenvironment associated with the EMT process. Genes mapped on the network were obtained from differentially expressed genes in the tumor buds and the microenvironment. The image was drawn with yEd graph editor (http://www.yworks.com/en/products/yfiles/yed/).

**Figure 7:**
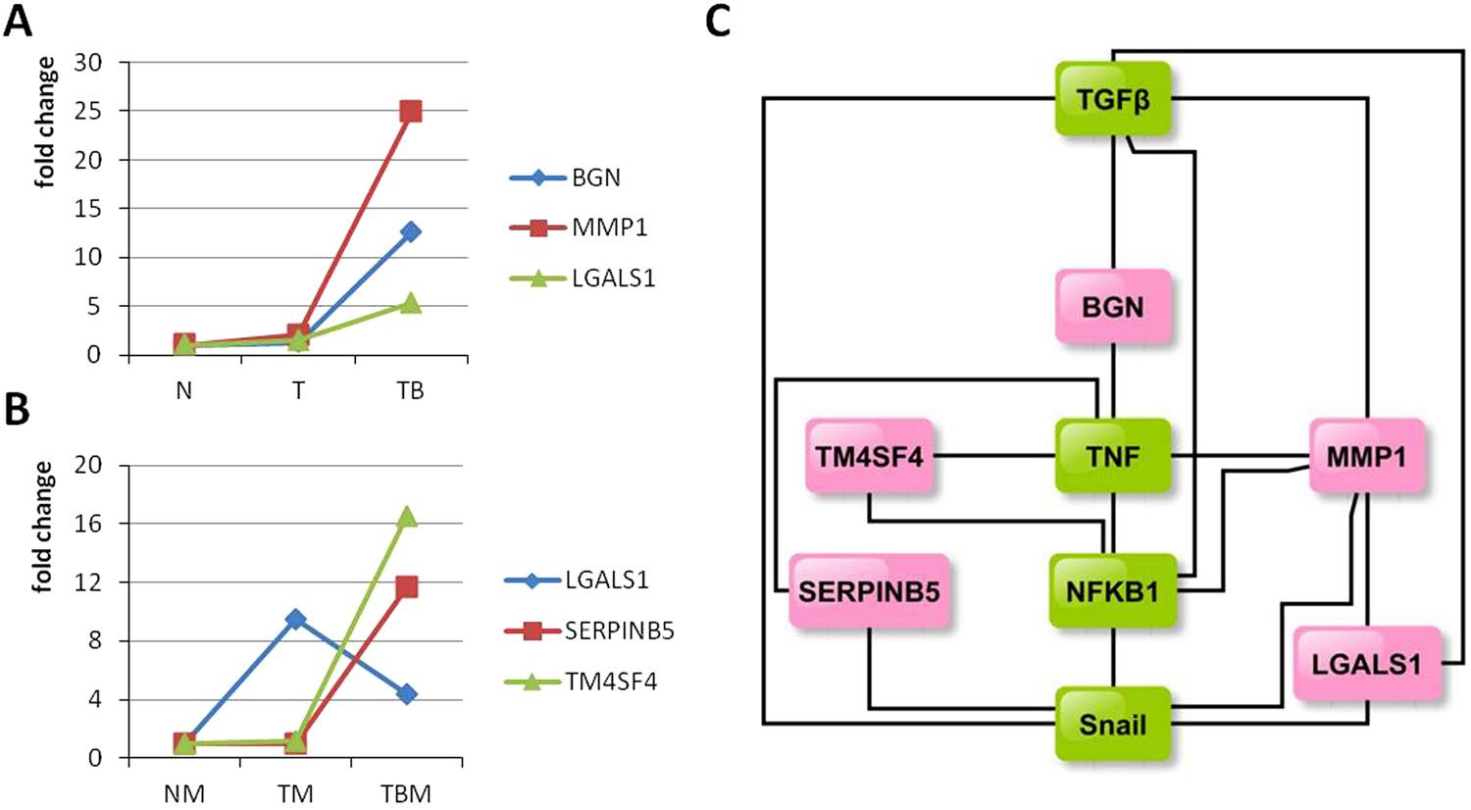
The regulatory relationships of candidate genes with the integrated pathway of TGFβ/Snail with TNFα/NFκB. (**A**) The expression levels of BGN, MMP1, and LGALS1 in IEC (N), CCPT (T), and tumor buds (TB). BGN, MMP1, and LGALS1 were upregulated in tumor bud cells. (**B**) The expression of LGALS1, SERPINB5, and TM4SF4 in the stromal cells of IEC (NM), CCPT (TM), and tumor buds (TBM). SERPINB5 and TM4SF4 were upregulated in TBM whereas LGALS1was downregulated, compared with the expression in NM and TM. (**C**) The candidate genes were functionally involved in the integrated pathway of TGFβ/Snail with TNFα/NFκB.

In support of the microarray and bioinformatic analysis data, and to uncover the essential transcription factors involved in the crosstalk between cancer cells and their microenvironment during the EMT process, we constructed an interactive-regulatory signaling network of EMT (Fig. 6). Among these differentially expressed genes mapped on the network, genes including *BGN, MMP1, LGALS1, SERPINB5*, and *TM4SF4* attracted our attention. In the tumor buds, *BGN, MMP1*, and *LGALS1* were upregulated (Fig. 7A). However, in the stromal cells of the tumor buds, *LGALS1* was downregulated and candidate genes *SERPINB5* and *TM4SF4* were upregulated, as compared with the control (Fig. 7B). To determine what type of signaling pathway the five candidate genes might affect during the EMT process, we constructed a regulatory network of *BGN, MMP1, LGALS1, SERPINB5*, and *TM4SF4*, on the basis of experimental literature and the KEGG database. Unexpectedly, all these genes participated in the integrated pathway of TGFβ/Snail and TNFα/NFκB (Fig. 7C). On the basis of the above results, we speculated that the integrated pathway of TGFβ/Snail with TNFα/NFκB might play a crucial role in the crosstalk of cancer cells with the microenvironment during the EMT process.

### Survival analysis of candidate genes in the CRC specimens

**Figure 8:**
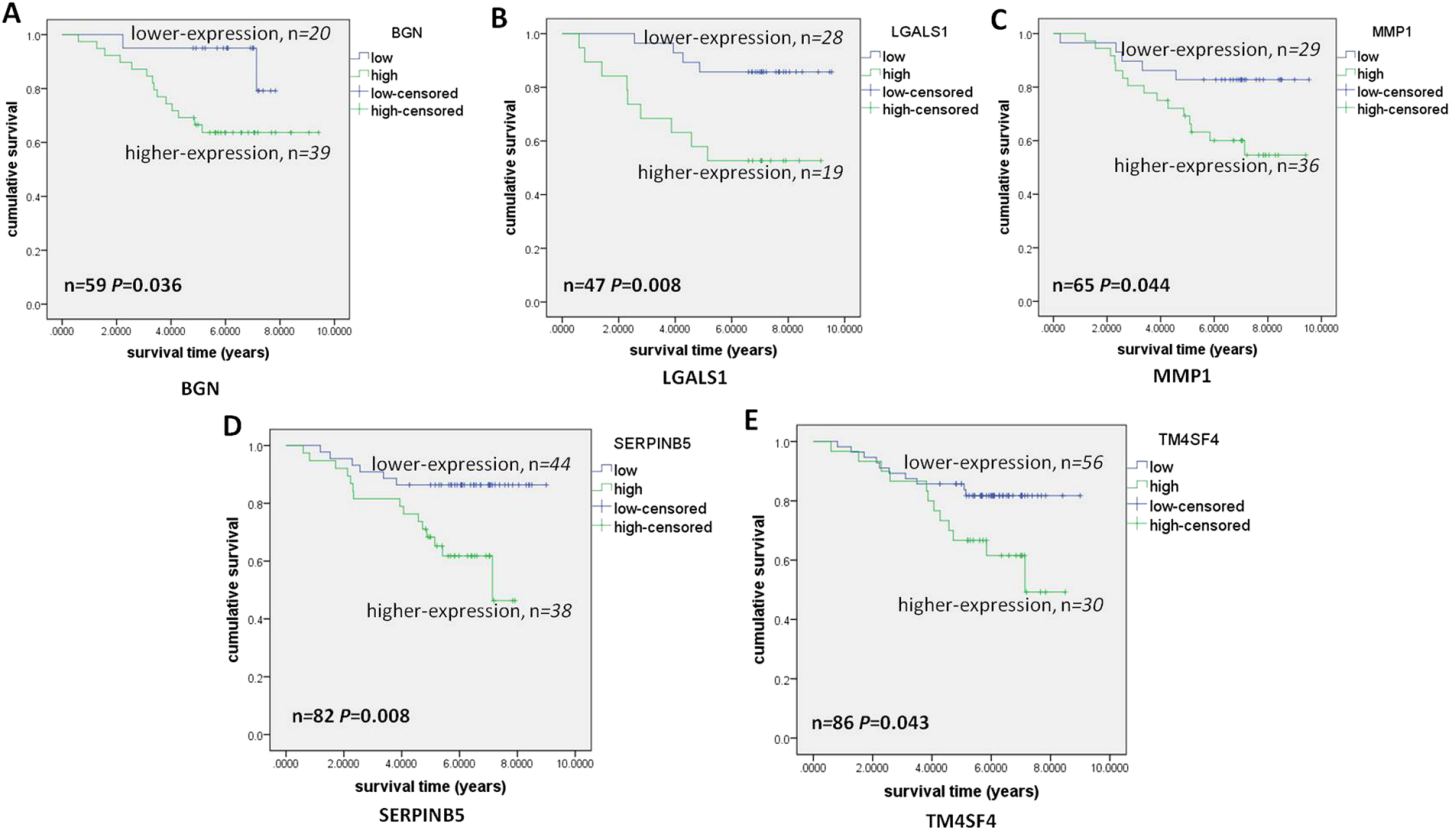
Kaplan-Meier analysis. (**A**) Survival analysis for the CRC patient specimens (n = 59). The patients with higher expression of *BGN* had poorer survival than those with lower expression of *BGN* (*P* = 0.036). (**B**) Survival analysis for the CRC patient specimens (n = 47). Kaplan-Meier univariate analysis demonstrated a significantly decreased survival probability in patients with higher *LGALS1* levels (*P* = 0.008). (**C**) Survival analysis for the CRC patient specimens (n = 65). Kaplan-Meier analysis demonstrated that patients with higher *MMP1* expression in CRC had poorer survival than those with low *MMP1* expression (*P* = 0.044). (**D**) Survival analysis for the CRC patient specimens (n = 82). The patients with high *SERPINB5* expression showed a decreased in survival probability compared with those with low *SERPINB5* expression (*P* = 0.008). (**E**), Survival analysis for the CRC patient specimens (n = 86). Kaplan-Meier analysis demonstrated a significantly decreased survival probability in patients with higher *TM4SF4* levels (*P* = 0.043, log-rank test).

The candidate genes including *BGN*, *MMP1*, *LGALS1*, *SERPINB5*, and *TM4SF4* probably correlated with cancer development and the EMT program mediated by the integrated pathway of TGFβ/Snail with TNFα/NFκB. The correlation of the candidate genes with the survival of CRC patients was assessed in the specimens. Total RNA was carefully isolated from the tumor cells of the CRC specimens and was reverse-transcribed to cDNA. Then, RT-PCR was performed to detect the expression levels of the candidate genes (*GAPDH* served as a housekeeping gene). We performed survival analysis based on the expression of the candidate genes with the Kaplan-Meier univariate method and the cutoff expression values of *BGN, MMP1, LGALS1, SERPINB5*, and *TM4SF4*, which distinguished the high and low expression, were defined by ROC curve (see Supplementary Figure S2). Kaplan-Meier analysis showed that high-levels of *BGN* (*P* = 0.036) and *MMP1* (*P* = 0.044) were significantly associated with poorer survival of the CRC patients (Fig. 8A,C). In addition, *BGN* and *MMP1* were both upregulated in the epithelial tumor cells. *LGALS1* (*P* = 0.008) was upregulated in the epithelium but downregulated in the mesenchymal cells and correlated with a poorer prognosis in the CRC patients with higher expression of the protein (Fig. 8B). In addition, the candidate genes *SERPINB5* (*P* = 0.008) and *TM4SF4* (*P* = 0.043), which were upregulated in the mesenchymal tumor cells, correlated with a significantly decreased survival potential in patients with higher expression levels (Fig. 8D,E). The results of the survival analysis verified that the overexpression of the five candidate genes that were mapped to the EMT-related TGFβ/Snail and TNFα/NFκB pathways correlated with low overall survival of patients with CRC. To validate the prognostic impacts of these five candidate genes, we collected clinical datasets from The Cancer Genome Atlas (TCGA), and performed survival analysis with the Kaplan-Meier univariate method. ROC curve was used to distinguish the high and low expression of candidate genes. It showed that higher expression of *BGN* (*P* = 0.001), *LGALS1* (*P* = 0.029) and *TM4SF4* (*P* = 0.001) predicted poorer CRC prognosis, which was concordant with our results. And high expression levels of *MMP1* (*P* = 0.716) and *SERPINB5* (*P* = 0.084) had no influence on clinical outcome (see Supplementary Figure S3).

## Discussion

Our study perform the first investigation of the interactive regulatory mechanism of cancer cells and their microenvironment during the EMT program with the model of tumor budding and the surrounding stromal components. Using classic and potential EMT biomarkers from cancer cells and their microenvironment, we have constructed a tumor-stroma crosstalk network of the EMT for the first time, on which five potential EMT and prognosis-related markers are mapped. We discovered that the five candidate genes including *BGN, MMP1, LGALS1, SERPINB5*, and *TM4SF4*, enriched in the integrated pathway of TGFβ/Snail with TNFα/NFκB, and these five candidate genes all predicted poorer prognosis when they were highly expressed. And *BGN* and *TM4SF4* are, for the first time, confirmed to have effects on CRC prognosis. On the basis of the above results, we come to a conclusion that the integrated pathway of TGFβ/Snail with TNFα/NFκB probably facilitates the tumor-stroma crosstalk during the EMT process and CRC prognosis.

The validity of our approach was supported by the identification of effective epithelial and mesenchymal markers (Fig. 5C). For example, the epithelial markers CDH1 and OCLN are down-regulated in tumor buds compared to the cancer cells in the center of the primary tumor, and up-regulated in the stroma of tumor budding compared to the stroma of regular cancer cells. Moreover, the mesenchymal markers FN1 is up-regulated in tumor buds compared to the regular cancer cells, and down-regulated in the stroma of tumor buddings compared to the stroma of regular cancer cells. This implies that the tumor buds have characteristics of mesenchymal cells and the stromal cells surrounding the tumor buds have part of epithelial characteristics, which indicate the communication between tumor budding and the microenvironment during EMT process. The identification of classical EMT regulators is also supported by relevant literature (Fig. 5B)^18, 19^.

Our approach for gene selection is supported by results from other studies. For example, IL6 was found to be upregulated both in the tumor buds and in the microenvironment (Fig. 5E) and to be located in the interactive-regulatory signaling network of EMT (Fig. 6). IL6 is usually secreted by activated stromal cells and is also found to be conditionally secreted by cancer cells^20^. The inflammatory cytokine, IL6, is capable of inducing EMT in various tumors through the IL6/STAT3 pathway^4^. Another study by Zhu and colleagues has demonstrated the interaction between gastric cancer-derived mesenchymal stem cells (GC-MSCs) and neutrophils *via* the IL6/STAT3 axis. The authors have found that the GC-MSCs induce the activation of neutrophils through the IL6-STAT3-ERK1/2 signaling axis, thereby promoting the migration of gastric cancer cells. In turn, neutrophils promote the differentiation of normal MSCs to GC-MSCs, thus forming a positive feedback loop for gastric cancer development, and this mechanism provides support for our analysis^21^.

Our study confirmed that, at higher expression levels, the candidate genes *BGN* (*P* = 0.036), *MMP1* (*P* = 0.044), *LGALS1* (*P* = 0.008), *SERPINB5* (*P* = 0.008), and *TM4SF4* (*P* = 0.043) from the tumor buds and the microenvironment signified a poorer prognosis for CRC patients, a result in accordance with the poor prognosis of tumor budding in CRC patients^14^. The overexpression of *MMP1* ^22^ and *LGALS1* ^23^ has been identified to correlate with poor prognosis of CRC patients, and upregulated *MMP1* and *LGALS1* induce the development of EMT in cancer^24, 25^. Prognostic analysis using CRC cohort from TCGA showed no prognostic significance of *MMP1* (see Supplementary Figure S3), whereas our result and another study have confirmed the prognostic significance of *MMP1* in CRC patients^22^. The candidate gene *BGN* is an ECM protein that belongs to a family of small leucine-rich proteoglycans^26^. *BGN* is upregulated in several types of cancer, such as CRC and pancreatic cancer^27, 28^. Ongoing research indicates that the dysregulation of *BGN* is associated with tumor cell invasion and migration^29–31^. The mechanism of the effects of *BGN* on tumor cell migration is likely to be mediated by the Rac1-mediated TGF-β signaling pathway, thus suggesting a role of *BGN* in the development of EMT^29^. Our study is the first to demonstrate the clinical significance of *BGN* for CRC patients, indicating a shorter survival time in the patients with a high level of *BGN*. *SERPINB5*, belonging to the serine protease inhibitor/non-inhibitor superfamily, is considered a tumor suppressor^32^. Our results confirmed that *SERPINB5* decreased the overall survival of the CRC patients, but the prognostic analysis using datasets from TCGA showed no prognostic significance of *SERPINB5* in CRC patients. The role of *SERPINB5* in cancer prognosis is paradoxical. *SERPINB5* has been related to good prognosis in lung, prostate, head and neck, and skin cancer, whereas in pancreatic cancer, it is associated with a poor prognosis. This contradiction is also reflected in CRC patients^33–35^. *TM4SF4* was, for the first time, confirmed to have an effect on the prognosis of CRC patients, but the biological function of TM4SF4 is mostly unknown.

We also identified the common pathway in which these five prognosis-related genes function (Fig. 7C). We presumed, on the basis of the above conclusions, that the integrated pathway of TGFβ/Snail with TNFα/NFκB might play a critical role in the crosstalk between cancer cells and their microenvironment in the development of EMT and CRC prognosis. The TGFβ/Snail pathway and the TNFα/NFκB pathway have been confirmed to be classical regulators in the EMT process^18, 36^. By regulating the expression levels and nuclear translocation of transcription factors in cancer cells and their microenvironment, both the TGFβ/Snail pathway and the TNFα/NFκB pathway participate in various stages of cancer development, including oncogenesis and cancer metastasis, as well as in the prognosis of cancer patients^37, 38^. Ongoing research indicates that TGFβ has close interactions with the inflammatory environment in the development of EMT, especially with the inflammatory factors TNFα and NFκB^39^. TGFβ, TNFα, and NFκB are capable of cooperatively mediating the development of EMT. In addition, Snail is at the crossroads of various stages of cancer, including EMT, formation of cancer cell stemness, metastasis and therapeutic resistance of tumors^18, 40^.

All of these reports support our finding that the integrated pathway of TGFβ/Snail with TNFα/NFκB might be the key to the interactions between cancer cells and their microenvironment and their effects on the EMT and CRC prognosis. These examples represent only the tip of the iceberg. The vast majority of the results generated from our analyses has never been evaluated before and will require external verification. Although remarkable progress has been made to investigate the underlying mechanism and numerous biomarkers have been verified, the understanding of the tumor-stroma coordinate regulation on the EMT is still rarely known. Recently, Takaaki and colleagues employ laser-captured tumor buds to study the effect of differentially expressed genes in tumor budding on the invasion of colon cancer^41^. By screening of clinical samples, they find that chemokines and EMT-related molecules may play critical roles in the invasion of colon cancer^41^. Our study focused on the use of bioinformatics and statistical analyses on large-scale transcriptional datasets, that were mined from tumor buds and the surrounding stromal cells to study the mechanism of the interactive regulations of cancer cell and the microenvironment as it pertained to EMT and cancer prognosis. We obtained numerous differentially expressed genes of tumor budding and the microenvironment, especially those mapped on the EMT regulatory network (Fig. 6), that could lay a foundation for the discovery of more EMT and prognosis markers. And our study also confirmed that the five candidate genes including *BGN, LGALS1, MMP1, SERPINB5*, and *TM4SF4* from tumor budding and the microenvironment signified a poorer prognosis for CRC patients when they were highly expressed. Meantime, these five candidate genes participated in the integrated pathway of TGFβ/Snail with TNFα/NFκB, thus suggesting that the integrated pathway of TGFβ/Snail with TNFα/NFκB might be the key to facilitate the tumor-stroma interaction during the EMT process and CRC prognosis. The true value of our work will arise from the use of these data and analyses to drive future mechanistic studies in pre-clinical cancer models to determine the cooperative regulation of the EMT program and cancer prognosis by cancer cell and the microenvironment and in building the foundation for the discovery of more effective therapeutic targets for CRC treatment.

## Methods

### Study subjects for laser capture microdissection and frozen sections

This study was approved by Zhejiang University’s School of Medicine and carried out in accordance with the approved guidelines and regulations. All experimental protocols were approved by Zhejiang University’s School of Medicine. All participants provided written, informed consent for participation in this study, and the ethics committee of Zhejiang University’s School of Medicine approved the study. Seven CRC specimens were collected from resected CRC samples from patients at the Sir Run Run Shaw Hospital, College of Medicine, Zhejiang University with informed consent and institutional IRB approval. The Samples were snap-frozen in liquid nitrogen and immediately stored at −80 °C. The cryotome (Thermo Fisher Scientific Inc., Waltham, MA, USA) was sterilized with isopropanol. The temperature for the cryotome chamber and sample holder was consistently maintained at −20 °C. Frozen 8-µm sections of the CRC samples were obtained and immediately stained using an LCM Frozen Section Staining Kit (Thermo Fisher Scientific Inc.).

### Laser capture microdissection and RNA isolation

LCM was performed at room temperature, and the relative humidity was maintained between 40–60%. Sections of the CRC specimens were prepared before microdissection. The tumor bud samples and stromal components were microdissected using an ArcturusXT™ microdissection system with a low-power infrared laser (Thermo Fisher Scientific Inc., Waltham, MA, USA). IEC and CCPT and their stromal components were dissected as controls. The captured cells were kept in 50 µL volume of extraction buffer and the assembly was immediately incubated for 30 min at 42 °C. After incubation, RNA from the captured cells was extracted using an RNA Isolation Kit (Thermo Fisher Scientific Inc.). The protocols can be viewed at https://tools.thermofisher.com/content/sfs/manuals/1268200.pdf.

### RNA amplification and microarray expression profile

Total RNA was amplified, labeled and purified using the Ovation RNA amplification system (Nugen Technologies Inc., San Carlos, CA, USA). Microarray analysis was performed on Affymetrix GeneChip Human Genome U133 plus 2.0 microarray (Affymetrix Inc, Santa Clara, CA, USA) at the microarray core facility of Shanghai Biotechnology Corporation.

### Bioinformatics summary

We used *t*-tests for the differential analysis of the expressed genes (*P* < 0.05). The *p*-values were adjusted using the Benjamini-Hochberg false discovery rate (FDR)^42^. FDR less than 0.05 were considered significant. DAVID 6.7 was used to execute the functional annotation (http://david.abcc.ncifcrf.gov/) and gene pathway enrichment was derived from the KEGG database (http://www.genome.jp/kegg/). Heatmap and Tree View were plotted using MATLAB with a matrix input of the gene expression values.

### Module analysis

WGCNA was performed using R (www.r-project.org). For WGCNA analyses, IEC, CCPT and tumor buds were analyzed separately. The absolute values of the Pearson correlation coefficients were calculated for all pairwise comparisons of gene-expression values across the three groups of samples. The correlation matrix for each species was then transformed into a matrix of connection strengths, using a power function (connection strength = |correlation | b), which resulted in a “weighted” network. To make meaningful comparisons across data sets, a power of b = 10 was chosen for all analyses. The function TOMdist1 computed the dissimilarity on the basis of the topological overlap matrix. To group the nodes with high topological overlap into modules (clusters), we used average linkage hierarchical clustering coupled with the TOM distance measure. We chose a height cutoff with a threshold of 0.995 to derive the clusters.

### Quantification of gene expression with NanoString nCounter and Codeset design

Direct digital cDNA analysis of gene expression was performed using a custom set of oligonucleotides synthetized by NanoString Technologies (The target region of candidate genes can be viewed in Supplementary Table S4 (NanoString Technologies, Inc., WA, USA). Hybridization and analysis were performed using a Prep Station and Digital Analyzer purchased from the company. Gene expression analysis was executed by nSovler2.0. The raw data were normalized with *t*-tests as well as the recommended standard from the NanoString data analysis guide (normalized by positive and negative controls, followed by global normalization). The probes were hybridized to 300 ng of total cDNA for 20 h at 65 °C and applied to the nCounter Preparation Station.

### Study subjects for ROC curve and Kaplan-Meier univariate analysis

This study was approved by Zhejiang University’s School of Medicine and was carried out in accordance with the approved guidelines and regulations. All experimental protocols were approved by Zhejiang University’s School of Medicine. All participants provided written, informed consent for participation in this study, and the ethics committee of Zhejiang University’s School of Medicine approved the study. The CRC specimens were acquired from Sir Run Run Shaw Hospital of Zhejiang University. The pathological diagnosis was evaluated by pathologists on the basis of biopsy reports, and patients who received radiation or chemo therapy were excluded. The specimens were obtained from patients with a diagnosis of CRC who underwent surgical resection with no chemotherapy or radiotherapy between 2003 and 2009 (see Supplementary Table S5).

### Quantitative RT-PCR

PCR was performed on a Real-time PCR Detection System (StepOnePlus; Thermo Fisher Scientific Inc.) with the following protocol: 95 °C for 1 min, followed by 40 cycles of 95 °C for 15 s, 60 °C for 15 s, and 72 °C for 45 s to detect the expression levels of *GAPDH, BGN, LGALS1, MMP1, SERPINB5* and *TM4SF4*. The primer sequences of five candidate genes were listed in Supplementary Table S6. *GAPDH* was used as an internal control. The 2^−ΔΔCT^ value was calculated for each sample and normalized to *GAPDH*. All RT-PCR results were representative of three independent experiments.

### Statistical analyses for ROC curve and Kaplan-Meier univariate analysis

Statistical analyses were performed with SPSS (SPSS 20.0; IBM Corp., New York, USA). Univariate survival analysis was performed and survival curves were drawn using the Kaplan-Meier method. The differences between the curves were tested using a log-rank test. A significant difference was identified at *P* < 0.05 (two-tailed).

## Electronic supplementary material

Supplementary Figure S1-3,Supplementary Table S1-6
