# Supplementary material for: The integrated pathway of TGFβ/Snail with TNFα/NFκB may facilitate the tumor-stroma interaction in the EMT process and colorectal cancer prognosis
Source: Sci Rep. 2017 Jul 7;7:4915. doi: 10.1038/s41598-017-05280-6 (PMC5501852; doi:10.1038/s41598-017-05280-6)
Supplement: Supplementary file 1 — Supplementary Figure S1-3,Supplementary Table S1-6 [file 41598_2017_5280_MOESM1_ESM.pdf]

The integrated pathway of TGFβ/Snail with TNFα/NFκB may facilitate the tumor-sroma interaction in the EMT process and colorectal cancer prognosis

Hui Li[1,2], Anjing Zhong[1,2], Si Li[1,2], Xianwen Meng[3], Xue Wang[4], Fangying Xu[1,2], Maode Lai[1,2]

[1]Department of Pathology, School of Medicine, Zhejiang University, Hangzhou310058, China.

[2]Key Laboratory of Disease Proteomics of Zhejiang Province, Hangzhou 310058, China.

[3]Department of Bioinformatics, State Key Laboratory of Plant Physiology and Biochemistry, College of Life Sciences, Zhejiang University, Hangzhou 310058, China.

[4]China Pharmaceutical University, Nanjing 320100, China.

Corresponding author: Maode Lai, Department of Pathology, Zhejiang University School of Medicine, Hangzhou 310058, PR China. Tel: + 86-571-88208200; Fax: +86-571-88208197; Email: lmp@zju.edu.cn.

Supplementary Figure S1. The principal component analysis (PCA) of IEC, CCPT, tumor budding, and their surrounding stromal components.

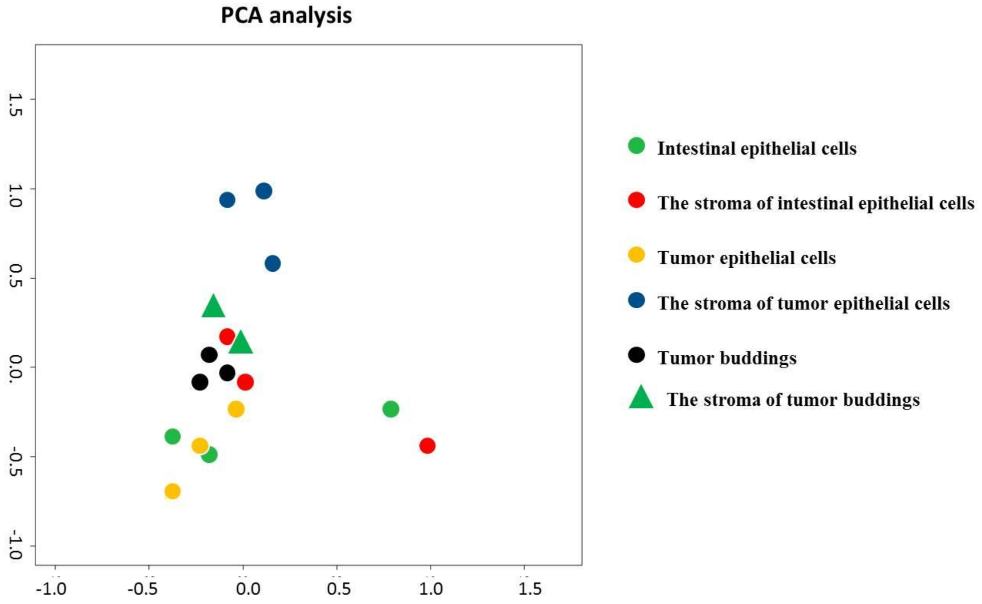

Supplementary Figure S2. Receiver operating characteristic (ROC) curve analysis.

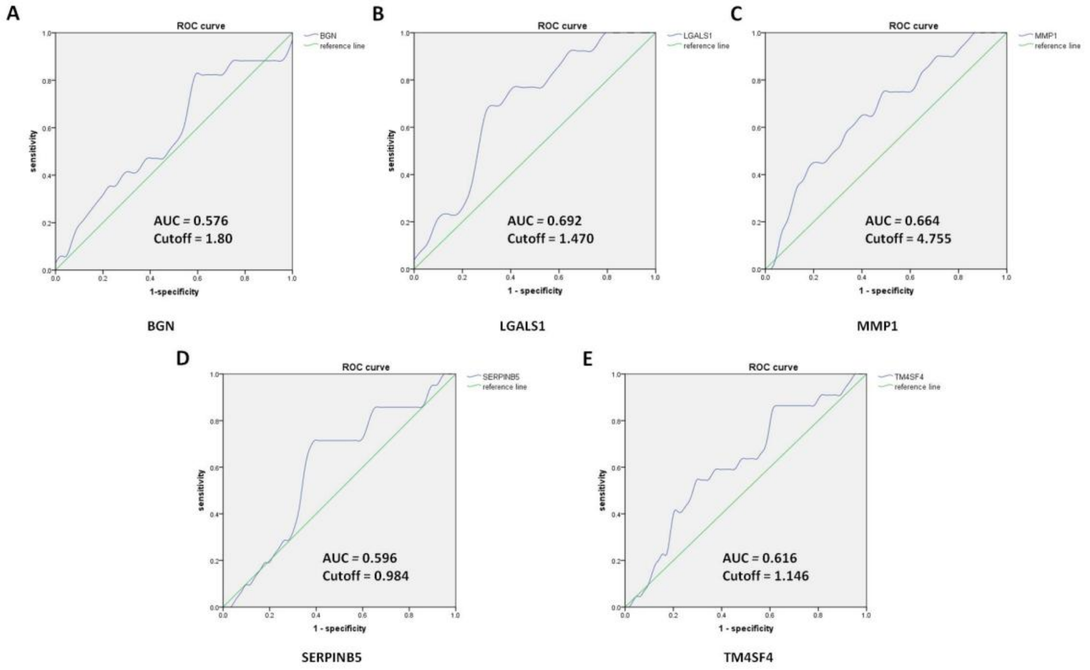

Supplementary Figure S2. ROC curve analysis. ROC curve analysis was used to determine the best cutoff expression value of five candidate genes for distinguishing the high and low expression of *BGN*, *LGALS1*, *MMP1*, *SERPINB5* and *TM4SF4*. AUC represents the Area under roc Curve. The cutoff was specified from the ROC curve using the optimal intersection of specificity and sensitivity. (**A**) ROC curve for *BGN*. (**B**) ROC curve for *LGALS1*. (**C**) ROC curve for *MMP1*. (**D**) ROC curve for *SERPINB5*. (**E**) ROC curve for *TM4SF4*.

Supplementary Figure S3.

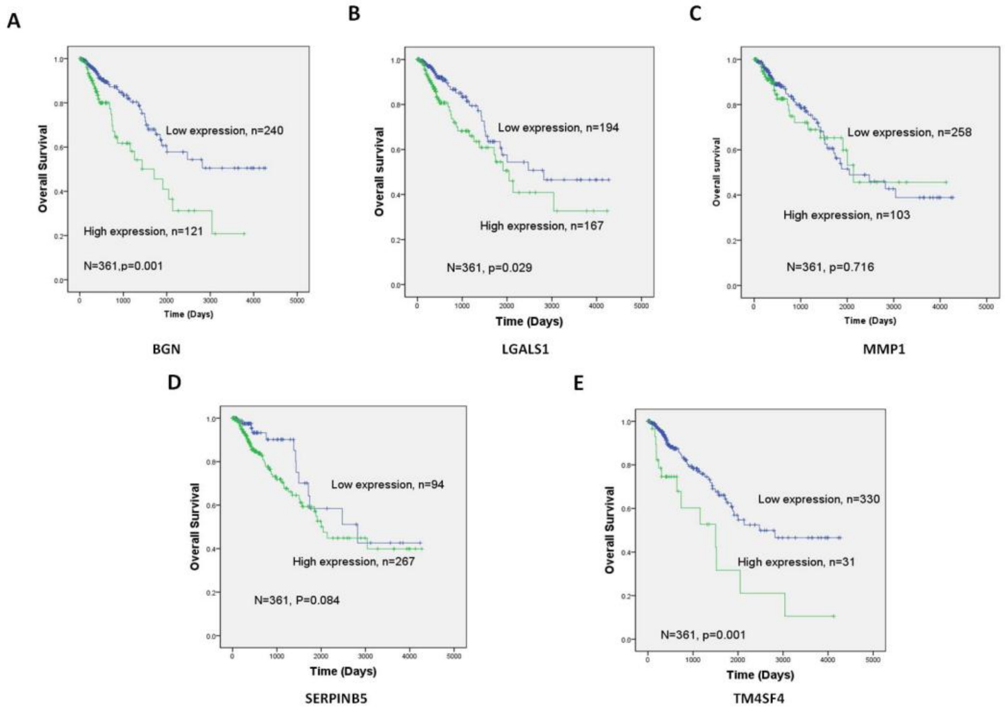

Supplementary Figure S3. Kaplan-Meier analysis using datasets from The Cancer Genome Atlas (TCGA). (**A**) Survival analysis for the CRC patient specimens (n = 361). The patients with higher expression of *BGN* had poorer survival (*P* = 0.001). (**B**) Survival analysis for the CRC patient specimens (n = 361). The patients with higher expression of *LGALS1* had poorer survival (*P* = 0.029). (**C**) Survival analysis for the CRC patient specimens (n = 361). Prognostic analysis using CRC cohort from TCGA showed no prognostic significance of *MMP1* (*P* = 0.716). (**D**) Survival analysis for the CRC patient specimens (n = 361). Prognostic analysis using CRC cohort from TCGA showed no prognostic significance of *SERPINB5* (*P* = 0.084). (**E**), Survival analysis for the CRC patient specimens (n = 361). Kaplan-Meier analysis demonstrated a significantly decreased survival probability in patients with higher *TM4SF4* levels (*P* = 0.001, log-rank test)

Supplementary Table S1. Specific gene sets of tumor budding

Table S1. Specific gene sets of tumor budding				
**brown**	**green**	**magenta**	**pink**	**purple**
AASS	*A1CF*	*ANKRD36BP2*	*ADH1B*	*CENPF*
ABHD5	*ACOX1*	*ARHGEF9*	*BCAS1*	*CMAH*
ACTA2	*AHNAK2*	*BRI3BP*	*BTBD19*	*DIO2*
ACTC1	*AKAP12*	*C6orf89*	*C6orf136*	*ETFDH*
ADCK1	*ALPK1*	*CHDH*	*CCNF*	*FNDC1*
AGFG2	*AP1M1*	*COL8A1*	*CD47*	*GOLGA2B*
AKAP2	*APOL6*	*CTSK*	*CDC42*	*GPR171*
ANKRD6	*ATPAF1*	*FAM49B*	*CPM*	*GRIN2C*
AOC3	*C1D*	*GAB1*	*CTNNB1*	*HECW1*
ARHGAP5	*C2orf15*	*HIST1H1D*	*CXCL1*	*HOXD3*
ARMCX2	*C5AR1*	*IGLV1-44*	*DDR2*	*IL8*
ASPN	*CA12*	*ITM2C*	*FBLN1*	*ITGB1*
ATP10A	*CA4*	*IWS1*	*FBN2*	*KIAA1267*
BASP1	*CARD16*	*LTBP2*	*GGTLC1*	*KRT17*
BMP7	*CASP4*	*MRPL35*	*GNG2*	*LDHD*
C14orf182	*CD274*	*MRPL37*	*GPC6*	*LGR4*
C17orf90	*CDX1*	*NEDD9*	*HMCN1*	*METTL8*
C1orf175	*CDX2*	*NR4A2*	*KIAA1549*	*MITF*
C2orf74	*CEACAM7*	*NUDT16*	*LIX1L*	*MRPL43*
C3orf14	*CFH*	*PKIB*	*MED28*	*NEDD4L*
C5orf4	*CLINT1*	*PREX1*	*MEF2C*	*PBRM1*
C8orf39	*CMAS*	*PRR15L*	*MVP*	*PPID*
CASD1	*COL10A1*	*PRRX1*	*MYO1A*	*QKI*
CAV1	*COL12A1*	*RAB31*	*NAV1*	*RAB15*
CAV2	*COL15A1*	*RFC2*	*OLFML2B*	*RRAS2*
CBLB	*COL3A1*	*RNF19B*	*OSBP2*	*SCFD2*
CCL28	*CP*	*RORA*	*PCDH7*	*SUV39H2*
CDHR5	*CYP2B6*	*SDC2*	*PLAC8*	*TBXA2R*
CDKN1C	*CYR61*	*SLC23A1*	*SCARA5*	*TSPAN7*
CHD2	*DACT1*	*SLC4A4*	*SIDT1*	*VPS53*
*PC1_var ＞ 0.90;density ＞ 0.70				

Supplementary Table S2. The biological process enrichment analysis on five gene sets of tumor budding

Table S2. The biological process enrichment analysis on five gene sets of tumor budding		
**Biological Process**	**Gene Count**	***P value***
GO:0040012~regulation of locomotion	14	5.29E-10
GO:0007155~cell adhesion	23	1.09E-09
GO:0022610~biological adhesion	23	1.12E-09
GO:0030334~regulation of cell migration	13	1.49E-09
GO:0006928~cell motion	19	2.54E-09
GO:0016477~cell migration	15	4.73E-09
GO:0051270~regulation of cell motion	13	6.76E-09
GO:0042127~regulation of cell proliferation	23	9.51E-09
GO:0048870~cell motility	15	1.83E-08
GO:0051674~localization of cell	15	1.83E-08
GO:0045944~positive regulation of transcription from RNA polymerase II promoter	16	2.71E-08
GO:0048545~response to steroid hormone stimulus	12	6.98E-08
GO:0007167~enzyme linked receptor protein signaling pathway	15	7.08E-08
GO:0009611~response to wounding	18	8.62E-08
GO:0009967~positive regulation of signal transduction	14	9.25E-08

Supplementary Table S3. KEGG pathway enrichment analysis on five gene sets of tumor budding

Table S3. KEGG pathway enrichment analysis on five gene sets of tumor budding		
**KEGG Pathway**	**Genes Count**	***P* value**
hsa04510:Focal adhesion	18	5.44E-10
hsa05200:Pathways in cancer	18	0.000000837
hsa04512:ECM-receptor interaction	9	0.0000131
hsa04350:TGF-beta signaling pathway	9	0.000017
hsa04520:Adherens junction	8	0.0000641
hsa05410:Hypertrophic cardiomyopathy (HCM)	8	0.000121
hsa04060:Cytokine-cytokine receptor interaction	12	0.000613
hsa05414:Dilated cardiomyopathy	7	0.00132273
hsa04514:Cell adhesion molecules (CAMs)	8	0.001757289
hsa04810:Regulation of actin cytoskeleton	10	0.002047359
hsa04144:Endocytosis	9	0.002906619
hsa05219:Bladder cancer	4	0.018104546
hsa05412:Arrhythmogenic right ventricular cardiomyopathy (ARVC)	5	0.018247224
hsa04640:Hematopoietic cell lineage	5	0.027359219
hsa04530:Tight junction	6	0.032694744
hsa05130:Pathogenic Escherichia coli infection	4	0.040074624
hsa04621:NOD-like receptor signaling pathway	4	0.049433604

Supplementary Table S4. Sequence-specific probes constructed for the analysis of 268 genes using nCounter system.

Table S4. Sequence-specific probes constructed for the analysis of 268 genes using the nCounter system.

Candidate genes	Refseq ID	Targeted region	Candidate genes	Refseq ID	Targeted region	Candidate genes	Refseq ID	Targeted region	Candidate genes	Refseq ID	Targeted region
ABCB1	NM_000927.3	3911-4010	ERBB2	NM_001005862.1	1256-1355	MET	NM_001127500.1	1926-2025	SCRIB	NM_182706.3	5018-5117
ABL1	NM_005157.3	3201-3300	EZH2	NM_004456.3	191-290	MFAP5	NM_003480.2	56-155	SDC1	NM_002997.4	2681-2780
ACOX1	NM_004035.5	2951-3050	FBLN1	NM_006487.2	966-1065	MGC40069	BC032242.1	439-538	SDC2	NM_002998.3	816-915
ACTA1	NM_001100.3	46-145	FBP1	NM_000507.3	591-690	MITF	NM_000248.3	3241-3340	SFI1	NM_001258326.1	845-944
ACTA2	NM_001613.1	646-745	FBXW9	NM_032301.2	1177-1276	MMP1	NM_002421.2	701-800	SHH	NM_000193.2	382-481
ACTB	NM_001101.2	1011-1110	FGFR1	NM_015850.2	1336-1435	MMP2	NM_004530.2	2361-2460	SLC7A11	NM_014331.3	637-736
ACTC1	NM_005159.4	2006-2105	FLT1	NM_002019.4	531-630	MSN	NM_002444.2	2631-2730	SLC9A1	NM_003047.3	1391-1490
ACTR2	NM_001005386.2	3205-3304	FMNL2	NM_052905.3	2151-2250	NANOG	NM_024865.2	1101-1200	SLIT2	NM_004787.1	2431-2530
ACTR3	NM_005721.3	781-880	FN1	NM_212482.1	1777-1876	NCL	NM_005381.2	1493-1592	SMAD2	NM_005901.4	181-280
AFAP1	NM_001134647.1	1389-1488	FOSL1	NM_005438.3	1087-1186	NFASC	NM_001005388.2	1176-1275	SMAD3	NM_005902.3	4221-4320
AGK	NM_018238.3	817-916	FUS	NM_004960.2	1871-1970	NFKB1	NM_003998.2	1676-1775	SMAD4	NM_005359.3	1371-1470
AKT1	NM_005163.2	1773-1872	FUT8	NM_178157.1	4-103	NIPBL	NM_133433.3	8194-8293	SMARCA2	NM_001289396.1	4889-4988
AKT2	NM_001626.2	1451-1550	GAB1	NM_002039.3	691-790	NOTCH1	NM_017617.3	736-835	SMURF2	NM_022739.3	2606-2705
ALCAM	NM_001627.3	790-889	GNG2	NM_053064.4	1791-1890	NOTCH3	NM_000435.2	1966-2065	SNAI1	NM_005985.2	64-163
ARHGAP5	NM_001173.2	2565-2664	GPR128	NM_032787.1	2496-2595	NRP2	NM_003872.2	806-905	SNAI2	NM_003068.3	741-840
ARHGEF7	NM_001113511.1	1276-1375	GRB7	NM_005310.2	1011-1110	NSD1	NM_022455.4	3141-3240	SOD2	NM_000636.2	641-740
AURKA	NM_003600.2	406-505	GRIN2C	NM_000835.3	2104-2203	NUAK1	NM_014840.2	6066-6165	SORBS1	NM_001034956.1	4006-4105
BCR	NM_004327.3	2376-2475	GTF3C4	NM_012204.2	2506-2605	OCLN	NM_002538.3	1976-2075	SORBS2	NM_001145675.1	653-752
BGN	NM_001711.3	1936-2035	GZMK	NM_002104.2	701-800	OLFML3	NM_020190.2	1526-1625	SOX4	NM_003107.2	3041-3140
BIN1	NM_004305.2	1836-1935	HCLS1	NM_005335.4	516-615	PAG1	NM_018440.3	4211-4310	SPARCL1	NM_004684.4	2266-2365
BMP6	NM_001718.2	1046-1145	HDAC3	NM_003883.3	353-452	PALLD	NM_001166108.1	5451-5550	SRC	NM_005417.3	1411-1510
BMP7	NM_001719.1	526-625	HGF	NM_000601.4	551-650	PARD3	NM_019619.2	1061-1160	SRP54	NM_003136.3	141-240
BMPER	NM_133468.4	477-576	HIF1A	NM_001530.2	1986-2085	PARD6A	NM_001037281.1	1162-1261	SS18	NM_001007559.1	2598-2697
C3	NM_000064.2	4397-4496	HLA-DRA	NM_019111.3	336-435	PAX8	XM_005263760.1	556-655	ST14	NM_021978.3	1406-1505
C5AR1	NM_001736.2	1196-1295	HMGA1	NM_145904.1	872-971	PDE8A	NM_001243137.1	1097-1196	STAT3	NM_139276.2	4536-4635
CAND1	NM_018448.3	2821-2920	HMGA2	NM_003484.1	329-428	PDGFA	NM_002607.5	1277-1376	STRADA	NM_153335.5	441-540
CAV1	NM_001753.3	435-534	HNMT	NM_001024074.1	251-350	PDGFRA	NM_006206.3	1926-2025	SUGT1	NM_001130912.1	1181-1280
CAV2	NM_198212.1	552-651	HNRNPD	NM_002138.3	695-794	PHLDB2	NM_001134437.1	3303-3402	TBL1XR1	NM_024665.4	916-1015
CCL4	NM_002984.2	36-135	HOXB1	NM_002144.3	439-538	PIK3CA	NM_006218.2	2446-2545	TBXA2R	NM_001060.3	386-485
CCR1	NM_001295.2	536-635	HSPA4	NM_002154.3	1226-1325	PIK3CB	NM_006219.1	2946-3045	TCF4	NM_001083962.1	3136-3235
CD44	NM_001001392.1	430-529	IGF2	NM_001127598.1	2418-2517	PLEKHF2	NM_024613.2	756-855	TCP1	NM_001008897.1	1439-1538
CD47	NM_001777.3	898-997	IGFBP7	NM_001553.1	691-790	POU5F1	NM_002701.4	1226-1325	TEK	NM_000459.3	1895-1994
CD74	NM_001025158.2	190-289	IGKC	BC067092.1	295-394	PRLR	NM_001204318.1	564-663	TGFB1	NM_000660.3	1261-1360
CD93	NM_012072.3	4271-4370	IL24	NM_181339.1	1017-1116	PROM1	NM_006017.1	926-1025	THBS1	NM_003246.2	3466-3565
CDC27	NM_001256.3	2551-2650	IL2RA	NM_000417.1	1001-1100	PTEN	NM_000314.4	1352-1451	THBS2	NM_003247.2	4461-4560
CDC42	NM_001039802.1	391-490	IL6	NM_000600.1	221-320	PTK2	NM_005607.3	1006-1105	THSD7A	NM_015204.2	4655-4754
CDH1	NM_004360.2	1231-1330	ILK	NM_004517.2	796-895	PTP4A3	NM_007079.2	1096-1195	TIAM1	NM_003253.2	2663-2762
CDH2	NM_001792.3	942-1041	INHBA	NM_002192.2	491-590	PTPRF	NM_002840.3	6311-6410	TJP1	NM_003257.3	6278-6377
CDH22	NM_021248.2	2227-2326	IQCG	NM_001134435.1	626-725	PTPRM	NM_002845.3	1681-1780	TLE6	NM_001143986.1	1677-1776
CFH	NM_001014975.2	703-802	ITGA11	NM_012211.3	651-750	PTPRS	NM_002850.3	1306-1405	TM4SF1	NM_014220.2	96-195
CLCA1	NM_001285.3	2706-2805	ITGA5	NM_002205.2	926-1025	PVRL3	NM_015480.2	926-1025	TMEM171	NM_173490.6	493-592
CLDN1	NM_021101.3	411-510	ITGA6	NM_000210.1	3066-3165	QKI	NM_006775.2	839-938	TNF	NM_000594.2	1011-1110
CLDN11	NM_001185056.1	1254-1353	ITGA8	NM_003638.1	1216-1315	QTRTD1	NM_024638.2	1401-1500	TPH1	NM_004179.1	336-435
CLDN16	NM_006580.2	446-545	ITGB1	NM_033666.2	2001-2100	RAB11B	NM_004218.3	515-614	TRAF3IP3	NM_025228.1	1472-1571
CRB1	NM_001193640.1	1375-1474	ITGB3	NM_000212.2	4486-4585	RAB34	NM_031934.5	1642-1741	TSPAN4	NM_001025234.1	1166-1265
CRB2	NM_173689.6	2093-2192	ITGB6	NM_000888.3	1091-1190	RANBP2	XM_005264006.1	4735-4834	TUB	NM_003320.4	1375-1474
CRB3	NM_139161.3	301-400	JAK3	NM_000215.2	1716-1815	RARB	NM_000965.3	2756-2855	TWIST1	NM_000474.3	394-493
CREB1	NM_134442.2	201-300	KIF11	NM_004523.3	383-482	RBPMS	NM_001008710.1	843-942	TXLNA	NM_175852.3	4127-4226
CSNK1A1	NM_001892.4	2054-2153	KIT	NM_000222.1	6-105	RBPMS2	NM_194272.1	443-542	UCHL5	NM_015984.2	318-417
CSNK2A1	NM_177559.2	1931-2030	KRAS	NM_004985.3	328-427	RDX	NM_002906.3	1971-2070	UPF1	NM_002911.3	5021-5120
CTNNB1	NM_001904.3	2266-2365	LAMB1	NM_002291.2	3121-3220	RFC2	NM_181471.1	836-935	VANGL1	NM_138959.2	721-820
CTNND1	NM_001085460.1	230-329	LASP1	NM_006148.2	3136-3235	RGS4	NM_005613.3	1536-1635	VASP	NM_003370.3	1501-1600
CTR9	NM_014633.3	1271-1370	LDOC1	NM_012317.2	967-1066	RGS5	NM_003617.2	2906-3005	VIM	NM_003380.2	695-794
CTSA	NM_001127695.1	1541-1640	LGALS1	NM_002305.3	61-160	RHOA	NM_001664.2	1231-1330	VSIG2	NM_014312.3	798-897
CTSK	NM_000396.2	416-515	LGR5	NM_003667.2	1469-1568	RHOJ	NM_020663.3	816-915	WAS	NM_000377.2	91-190
CXCL1	NM_001511.1	743-842	LPCAT3	NM_005768.5	510-609	RMDN1	NM_016033.2	991-1090	WASF1	NM_001024934.1	1426-1525
CXCL14	NM_004887.4	1126-1225	LRPPRC	NM_133259.3	3049-3148	RMDN3	NM_018145.1	2033-2132	WDR5	NM_017588.2	1781-1880
CYBA	NM_000101.2	15-114	LRRC8E	NM_025061.3	451-550	ROBO1	NM_002941.2	6396-6495	WDR77	NM_024102.2	1541-1640
DAPK2	NM_014326.3	754-853	LTBP1	NM_000627.2	156-255	RORA	NM_134261.2	1716-1815	WNK1	NM_018979.1	4531-4630
DCN	NM_001920.3	421-520	MAF	NM_001031804.2	6161-6260	ROS1	NM_002944.2	4251-4350	WNT5A	NM_003392.3	476-575
DSP	NM_001008844.1	6026-6125	MAP2	NM_031845.2	5171-5270	RPS21	NM_001024.3	131-230	ZBTB16	NM_006006.4	1586-1685
EGF	NM_001963.4	1023-1122	MAPK1	NM_138957.2	431-530	RRAS2	NM_001102669.2	1786-1885	ZEB1	NM_001128128.1	1451-1550
EGFR	NM_201282.1	361-460	MAPK10	NM_138981.2	451-550	RUNX1T1	NM_004349.2	1086-1185	ZEB2	NM_001171653.1	441-540
EHD2	NM_014601.2	1476-1575	MAPK8	NM_139047.1	836-935	S100A4	NM_002961.2	264-363	ZIK1	NM_001010879.2	561-660
EHMT2	NM_025256.5	2881-2980	MEF2C	NM_002397.3	2446-2545	S100A8	NM_002964.3	116-215	ZNF100	NM_173531.3	917-1016
EIF5B	NM_015904.3	3215-3314	MEF2D	NM_005920.2	3686-3785	SCAMP5	NM_001178111.1	686-785	ZNF462	NM_021224.4	3026-3125
EPCAM	NM_002354.1	416-515	MEIS1	NM_002398.2	2026-2125	SCN2A	NM_021007.2	6213-6312	ZNF655	NM_001083956.1	817-916

Supplementary Table S5. Clinical data of CRC specimens.

Table S5. Clinical data of CRC specimens					
	BGN (n = 59)	*LGALS1* (n = 47)	*MMP1* (n = 65)	*SERPINB5* (n = 82)	*TM4SF4* (n = 86)
**sex**					
male	31(52.5)	25(53.2)	37(56.9)	42(51.2)	39(45.3)
female	28(47.5)	22(46.8)	28(43.1)	40(48.8)	47(54.7)
**age(years)**					
＜60	21(35.6)	17(36.2)	16(24.6)	24(29.3)	27(31.4)
≥60	38(64.4)	30(63.8)	49(75.4)	58(70.7)	59(68.6)
median	63	63	64	67	66
**overall survival status**					
alive	43(72.9)	34(72.3)	45(69.2)	61(74.4)	64(74.4)
die	16(27.1)	13(27.7)	20(30.8)	21(25.6)	22(25.6)
**distant metastasis**					
negative	51(86.4)	37(78.7)	53(81.5)	70(85.4)	72(83.7)
positive	8(13.6)	10(21.3)	12(18.5)	12(14.6)	14(16.3)

Supplementary Table S6. The primer sequences for RT-PCR.

Table S6. The primer sequences of candidate genes for RT-PCR.		
**genes**	**sense**	**antisense**
BGN	CAAGGTGCCCAAGGGAGTGTT	CATTCAGGGTCTCAGGGAGGTCT
LGALS1	CCATCTCTCTCGGGTGGAGTCTT	TTGTTGCTGTCTTTGCCCAGGTT
MMP1	GGCTGAAAGTGACTGGGAAAC	CTTGGCAAATCTGGCGTGTAA
SERPINB5	GGTCTTTGTGCTCCTCGCTT	CACTGGTTTGGTGTCTGTCTTG
TM4SF4	AGCGATTTGCGATGTTCACC	CGGAGGTTTAAACGGGTCCA
GAPDH	ACCACAGTCCATGCCATCAC	TCCACCACCCTGTTGCTGTA
